# Microstructure and Mechanical Properties of Modern 11%Cr Heat-Resistant Steel Weld Joints

**DOI:** 10.3390/ma14123430

**Published:** 2021-06-21

**Authors:** Grzegorz Golański, Jacek Słania, Marek Sroka, Paweł Wieczorek, Michał Urzynicok, Ryszard Krawczyk

**Affiliations:** 1 Department of Materials Engineering, Czestochowa University of Technology, Armii Krajowej 19, 42-200 Częstochowa, Poland; grzegorz.golanski@pcz.pl (G.G.); wieczorek.pawel@pcz.pl (P.W.); 2 Faculty of Mechanical Engineering and Computer Science, Czestochowa University of Technology, Armii Krajowej 21, 42-200 Częstochowa, Poland; jacek_slania@poczta.onet.pl (J.S.); ryszard.krawczyk@pcz.pl (R.K.); 3 Department of Engineering Materials and Biomaterials, Silesian University of Technology, Konarskiego St. 18a, 44-100 Gliwice, Poland; 4 Zelkot, Nowy Dwór 8, 42-286 Koszęcin, Poland; mu@zelkot.pl

**Keywords:** Thor 115 steel, microstructure, mechanical properties, weld joints, heat resistant steel

## Abstract

In addition to good high-temperature creep resistance and adequate heat resistance, steels for the power industry must have, among other things, good weldability. Weldability of such steels is one of the criteria determining whether or not the material is suitable for applications in the power industry. Therefore, when materials such as martensitic steel Thor 115 (T115) are introduced into the modern power industry, the quality and properties of welded joints must be assessed. The paper presents the results of metallographic and mechanical investigations of T115 martensitic steel welded joints. The analysis was carried out on joints welded with two filler metals: WCrMo91 (No. 1) and EPRI P87 (No. 2). The scope of the investigations included: microstructural investigations carried out using optical, scanning and transmission electron microscopy and mechanical testing, i.e., Vickers microhardness and hardness measurement, static tensile test and impact test. The macro- and microstructural investigations revealed correct structure of the weld, without welding imperfections. The microstructural investigations of joint No. 1 revealed a typical structure of this type of joint, i.e., the martensitic structure with numerous precipitates, while in joint No. 2, the so-called Nernst’s layers and δ-ferrite patches were observed in the weld fusion zone as well as the heat affected zone (HAZ). The mechanical properties of the test joints met the requirements for the base material. A slight influence of the δ-ferrite patch on the strength properties of joint No. 2 was observed, and its negative effect on the impact energy of HAZ was visible.

## 1. Introduction

The construction of modern supercritical or ultra-supercritical boilers to reduce air emissions and fuel consumption required the use of modern creep-resistant materials, including but not limited to 9–12% Cr martensitic steels, for boiler pressure components. Martensitic steels are intended for, but not limited to, steam superheater components which are considered essential for a power unit [1,2]. However, the operating temperature of this group of materials is limited to 610–620 °C. It is related to the limited corrosion and oxidation resistance of 9% Cr steels and the microstructural instability of 12% Cr steels due to the transformation of metastable MX precipitates into Z-phase. The limited operating temperature of martensitic steels resulted in the introduction of austenitic steels in the power industry [2,3,4]. Due to their adverse physical properties and susceptibility to intergranular and stress corrosion, the creep-resistant austenitic steels give rise to a number of operational problems [5,6]. Therefore, research aimed at the development of new types of steel with ferritic matrix capable of operating at above 610 (620) °C is being carried out in numerous research centres.

One of these modern materials classified as 9–12% Cr martensitic steels is the steel marked Thor 115 (T115). The T115 steel was developed as a result of the modification of the chemical composition of the T/P91 steel by increasing the content of chromium by approximately 11% and optimising the content of molybdenum and niobium [7]. The increase in the content of chromium is to provide a better corrosion and oxidation resistance, whereas the rationalisation of the content of molybdenum and niobium is to limit the precipitation of adverse secondary phases, i.e., Laves phase and Z phase.

The suitability of a material for specific applications is determined by the set of performance properties—its high-temperature creep resistance and heat resistance, but also its satisfactory technological properties, such as weldability, are important here. Good weldability of new steels for the power industry is required and necessary to avoid the case relating to the T24 steel, which has not been commonly used in modern supercritical power units due to its limited weldability [8]. Thus, the analysis of weldability of the steels for the power industry is an essential part of the development of material characteristics for modern creep-resistant materials. The selection of the filler metal is an important issue in terms of the expected long-term service of welded joints in power units. The correct selection of the filler metal for welding determines whether the required welded joint properties are obtained or not. Filler metals used for welding should provide the weld with chemical composition and mechanical properties which are as close as possible to those of the base material. The preliminary tests carried out in Zelkot company [9] revealed good weldability and correct mechanical properties of joints made in T115 steel. Similar test results for T115 steel welded joints were presented in [10]. Nevertheless, the literature does not provide data on the properties and microstructure of welded joints made on T115 steel. Hence, this paper supplements the database and knowledge base in the area of assessment of T115 steel weldability with filler materials, i.e.: CrMo91 and EPRI P87. The analysis of the microstructure and selected mechanical properties of T115 steel welded joints was the paper’s purpose. The study of microstructure and mechanical properties covered both the parent material and the welded joints. The analysis of the test results obtained allowed the quality of the welded joints made on T115 steel using two substantially different filler materials to be assessed.

## 2. Materials and Methods

The material used in the investigations was welded joints made in T 115 steel. T115 steel with chemical composition as in Table 1 was delivered as tube specimens of 50.8 mm × 10.1 mm (external diameter × wall thickness). Table 2 presents the mechanical properties of T115 steel in the initial condition. The test welded joints were made by the TIG (tungsten inert gas, method 141) in 5G uphill position. The obtained joints were post-welding heat treated (PWHT) by annealing at 760 °C for 1 h after welding. 

**Figure 1:**
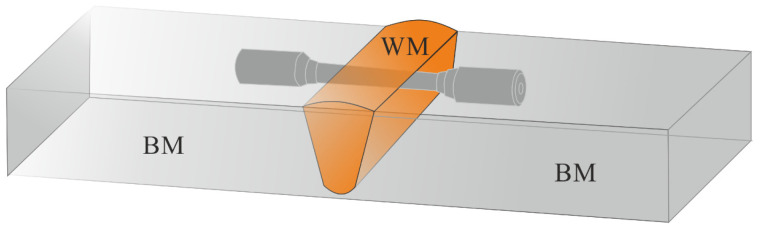
Schematic of the specimens prepared for mechanical tests from the welded joint.

The quality level B according to [11] was adopted as the assessment criterion. The analysis of the chemical composition of the base material was carried out using the SpectroLab (Kleve, Germany) spark spectrometer. The microstructure investigations were carried out with the Olympus SZ61 (Tokyo, Japan) optical microscope and the JEOL JSM-6610LV (Akishima, Japan) scanning electron microscope on prepared metallographic microsections etched with ferric chloride. Examining the microstructure and analysis of precipitates in the native material was carried out using the JEOL 2110plus (Akishima, Japan) transmission electron microscope with the use of thin foils. The Vickers hardness measurement was made with the FutureTech FV700 (Kangawa, Japan) hardness tester using an indenter load of 10 kG (98 N), whereas the microhardness measurement was made with the Shimadzu HMV-G20 (Kyoto, Japan) microhardness tester using a load 0.01 kG (0.098 N).The static tensile test was carried out on flat specimens with shoulders and an initial width of b_0_ = 12 mm using the Zwick Roel Z100 (Riedlingen, Germany) testing machine, and the impact test was carried out at room temperature on non-standard test Charpy V specimens with a reduced width of 7.5 mm. The mechanical investigations were carried out according to the guidelines in: hardness testing [12], Vickers hardness test [13], microhardness testing of welded joints [14], tensile testing at room temperature [15], tensile testing at elevated temperature [16], transverse tensile test [17], impact tests [18], Charpy pendulum impact test [19]. In each of the mechanical investigations, three samples were tested (Figure 1) and the average value of the three samples is shown. 

## 3. Filler Metal for Welding

The filler metal used for welding of T115 steel was CrMo91 (No. 1) and EPRI P87 (No. 2). Due to the absence of a filler metal dedicated to T115 steel welding, the steel producer recommends using the P91 steel-based material, i.e., CrMo91. This is probably due to the fact that T115 has weldability similar to that of P91 [9], comparable basic mechanical properties and availability of the filler metal and its price. 

The use of different materials in the construction of a power unit requires a number of dissimilar joints which, due to their reduced metallurgical stability, are considered to be most critical in the boiler design. Creep resistance of these joints is usually lower than in the designed one, which is related to two basic phenomena—differences in the linear thermal expansion of joined materials and the occurrence of diffusion processes in the fusion line area [20,21]. The developed EPRI P87 filler material significantly reduces the adverse effects of these phenomena on the performance of dissimilar joints. The thermal expansion coefficient of P87 is similar to the coefficients of P22 and P91/P92 steels. The trend towards carbon diffusion was also limited by reducing the chromium content to approximately 9% [22]. Chemical composition of the filler materials (according to the producer’s data) are shown in Table 3. Table 4 summarises their mechanical properties (according to the producer’s data).

## 4. Results and Discussion

### 4.1. Microstructure of T155 in Initial Condition

**Figure 2:**
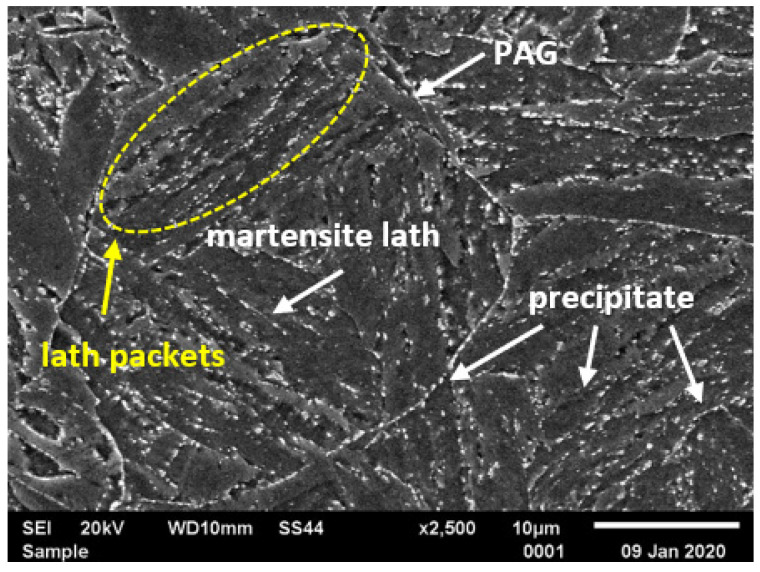
Microstructure of T115 steel in initial condition by scanning electron microscopy (SEM).

**Figure 3:**
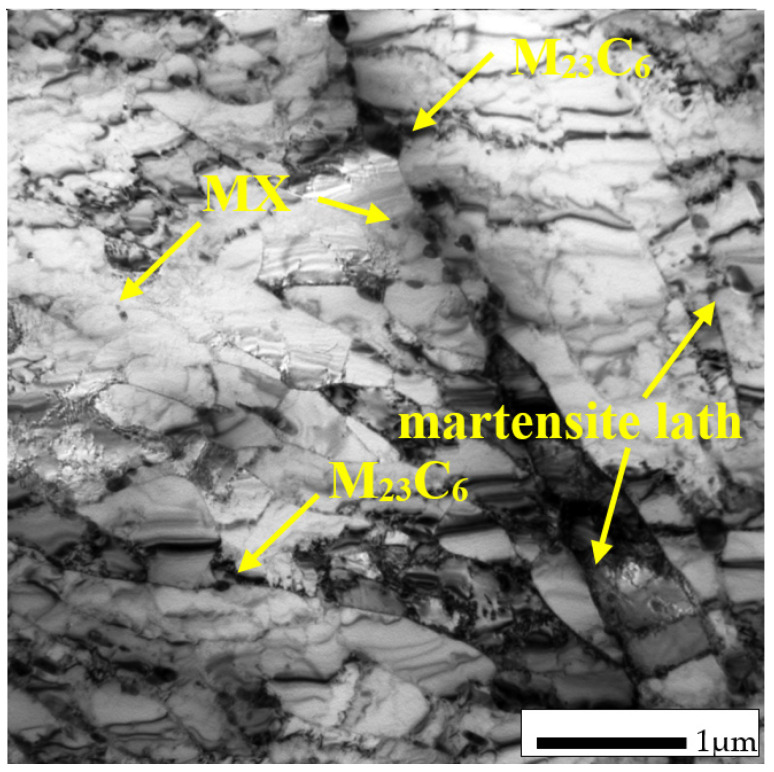
Microstructure of Thor 115 in initial condition by transmission electron microscopy (TEM).

**Figure 4:**
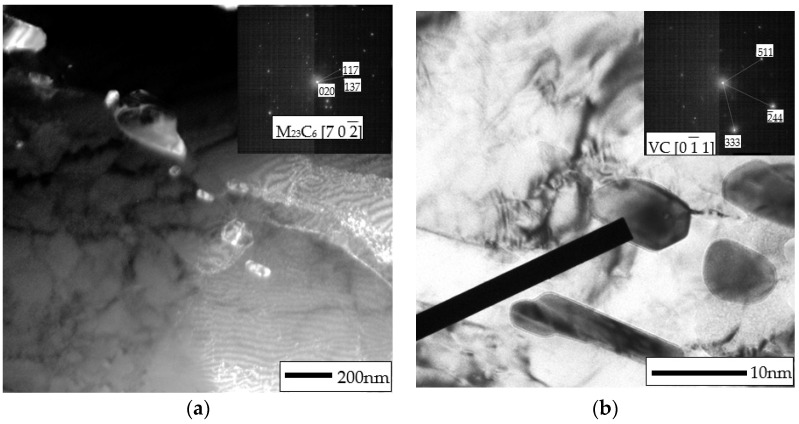
Precipitates in Thor 115 steel in initial condition: (**a**) M_23_C_6_; (**b**) MC.

Typical microstructures of T155 steel in the initial condition are presented in Figure 2 and Figure 3. In the initial condition, T115 steel had a structure of tempered martensite with numerous particles observed at the prior austenite grain (PAG) boundaries, martensite lath blocks, martensite lath boundaries and within laths/grains (Figure 2 and Figure 3). The lath martensite packets formed inside the PAG boundaries. The orientation and the size of the packets and lathes in the blocks were variable. The electron microscopic examinations revealed that the microstructure of tempered martensite had subgrain structure with a low density of dislocation inside the subgrains and numerous M_23_C_6_ and MX precipitates (Figure 3 and Figure 4). In the steel in the initial condition, two types of precipitate are observed: M_23_C_6_ carbides and MX (NbX, VX) precipitates were also observed for example in [1,23,24]. The M_23_C_6_ particles are chromium-rich precipitates [1,24,25]. Chromium-rich M_23_C_6_ carbides are the predominant type of precipitates in this group of steels. According to [22], they represent more than 90% of all precipitates in high-chromium steels. Particles of these precipitates are observed at the PAGs and martensite packet and lath boundaries, and single precipitates are also visible inside the laths. M_23_C_6_ carbides in 9–12%Cr steels play an important role by stabilising the lath structure of tempered martensite [1,26,27,28].

In turn, the MX particles (niobium and/or vanadium-rich precipitates) can be observed within the martensite laths, and part of them is precipitated at the dislocations. In the matrix, these precipitates are distributed inhomogeneously. Unlike M_23_C_6_ carbides, MX precipitates in 9%Cr steels are characterised by low coarsening rate. High thermal stability of these precipitates and coherent (semi-coherent) interfaces with the matrix cause that, despite their low volume fraction (approximately 0.020–0.025), these precipitates develop a strong precipitation hardening of steel and are an effective barrier to the free dislocation movement by the pinning effect [1,4].

### 4.2. Microstructure of T155 Heat-Affected Zone of Welded Joints

**Figure 5:**
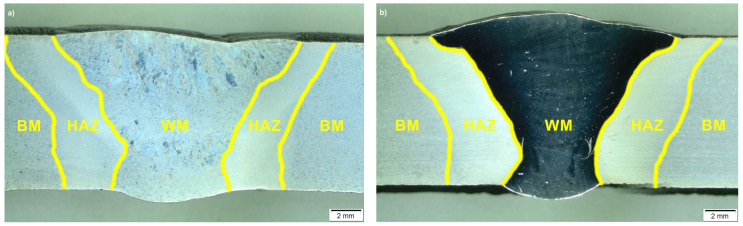
Macroscopic image of test joints: (**a**) joint No. 1; (**b**) joint No. 2; where: WM-weld; HAZ—heat affected zone; BM—basic material.

The macroscopic views of the T115 weld joints are shown in Figure 5. The effect of heat during the welding process changes the microstructure in the areas adjacent to the fusion line—this area is called the heat-affected zone (HAZ). Depending on the experience temperature, the following areas can be identified: coarse grained heat-affected zone (CGHAZ), fine-grained heat-affected zone (FGHAZ), and intercritical heat-affected zone (ICHAZ). The structure of the specific HAZ is determined by the peak of temperature.

**Figure 6:**
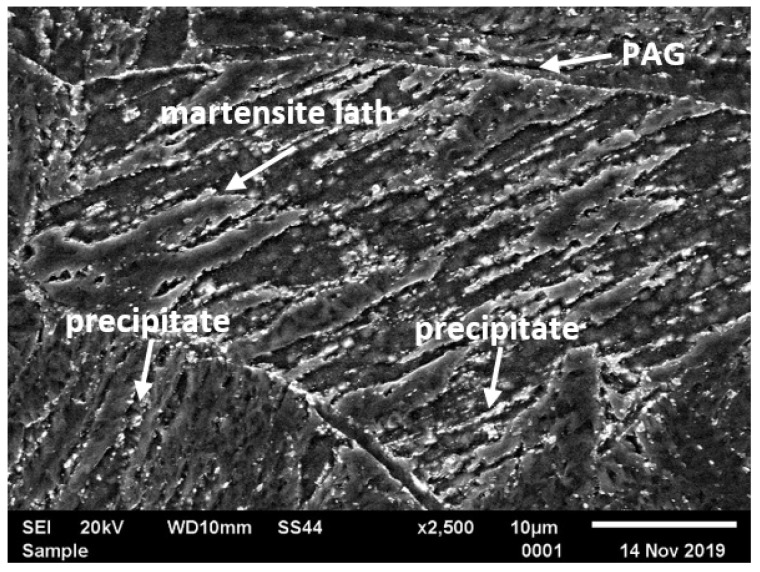
Microstructure of joint No. 1 in the vicinity of the fusion line—coarse grained heat-affected zone (CGHAZ).

In the CGHAZ near the fusion line, the temperature is significantly above transition temperature A_C3_ [29,30]. High peak temperature of the welding cycle in the zone contributes to dissolution of precipitates in the matrix, mainly M_23_C_6_ particles [29,31,32]. This dissolution results in disappearance of the boundary pinning effect and leads to the formation of coarse austenite grain (Figure 6). The measured size of PAGs in this area was 33 ± 8 µm, which corresponds to 7 according to the ASTM standard scale. In this area, the structure of tempered martensite with numerous precipitates of different morphologies is observed. The precipitates are arranged in a similar way as in the base material, i.e., at the PAGs, martensite lath boundaries and within the laths (Figure 2 and Figure 6). In the HAZ, similar to base material, the M_23_C_6_ precipitates are mainly observed at the boundaries/laths/blocks, while MX precipitates are observed inside the laths/subgrains [31,32]. The size of the prior austenite grain in martensitic steels is an important factor affecting the steel properties, such as: tensile strength, toughness, creep strength, as well as susceptibility to the damage mechanism, e.g., type IV cracking [31,33,34,35].

**Figure 7:**
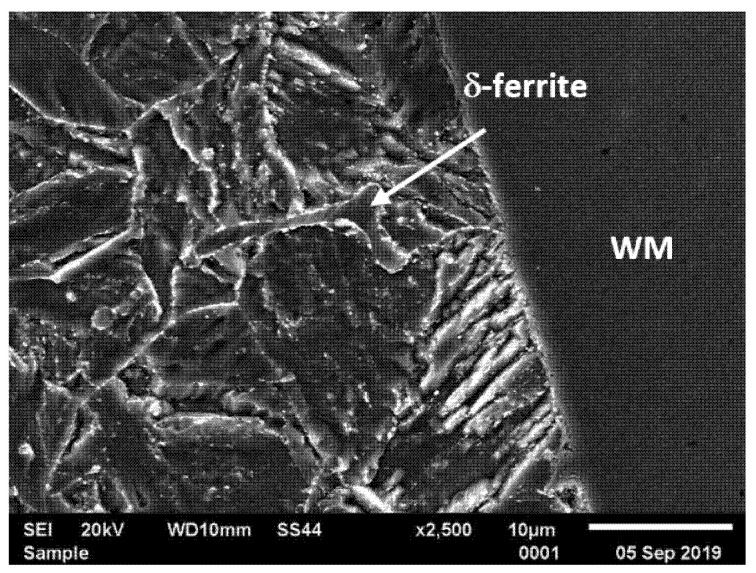
The δ-ferrite in the vicinity of the fusion line—CGHAZ, joint No. 2; WM—weld metal.

Similarly to joint No. 1, the coarse-grained martensitic microstructure with numerous precipitates are was also observed in the vicinity of the fusion line in joint No. 2. However, unlike in joint No. 1, the presence of a patch of δ-ferrite was observed at the grain boundaries in this area (Figure 7). The calculated volume fraction of δ-ferrite in this area of joint No. 2 was 1.54%. 

The susceptibility to possible precipitation of δ-ferrite near weld fusion zone can be determined for example by using the following equations [36]:

$$\begin{array}{c} Cr_{eq}=\%\mathrm{Cr}+6\%\mathrm{Si}+4\%\mathrm{Mo}+1.5\%W+11\%V+5\%\mathrm{Nb}+12\%\mathrm{Al}+8\%\mathrm{Ti}-40\%C-2\%\mathrm{Mn}-4\%\mathrm{Ni}\\ \;\;\;\;\;\;-2\%\mathrm{Co}-30\%N-\%\mathrm{Cu} \end{array}$$

$$\begin{array}{c} \mathrm{Kaltenhauser}\;\mathrm{ferrite}\;\mathrm{fator}\;\left(\mathrm{FF}\right)\\ \;\;\;\;\;\;=\;\%\mathrm{Cr}+6\%\mathrm{Si}+4\%\mathrm{Mo}+8\%\mathrm{Ti}+2\%\mathrm{Al}+4\%\mathrm{Nb}-2\%\mathrm{Mn}-4\%\mathrm{Ni}-40\%C-40\%N \end{array}$$

For structure without δ-ferrite, the value of Cr_eq_ and FF parameters should be lower than 10 and 8, respectively. For the test steel, the value of Cr_eq_ and FF parameters was 11.88 and 9.18, respectively. This indicates a tendency to the formation of δ-ferrite, which will grow if the content of ferrite-forming elements (mainly Cr, and also Mo) in the fusion line area increases. 

According to [31,33], the presence of δ-ferrite in this area of the welded joint may also be due to the composition of the base metal and filler material, wide solidification range, high heat input as well as cooling rate. In turn, according to [37], high heat input during the multipass welding process led to slow cooling rate and affected the formation of δ-ferrite in the area near the fusion line. 

The interior of the δ-ferrite area are observed to be free from the precipitates, while the boundaries are decorated with carbide precipitates (Figure 7). Presence of the δ-ferrite patch in steel accelerated the recovery rate of martensite structure and growth rate of Laves phase and, therefore, reduced the creep and fatigue strength of steel [33].

**Figure 8:**
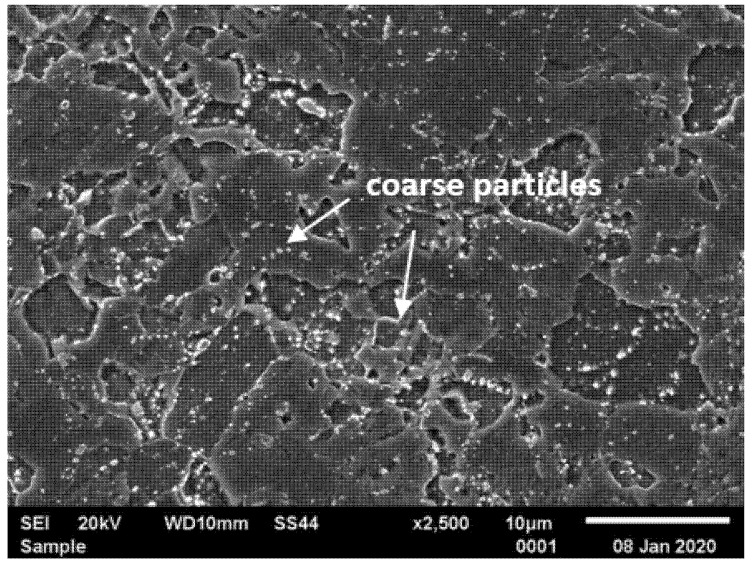
Microstructure of fine-grained heat-affected zone (FGHAZ)/intercritical heat-affected zone (ICHAZ) in T115 steel joint—joint No. 1.

**Figure 9:**
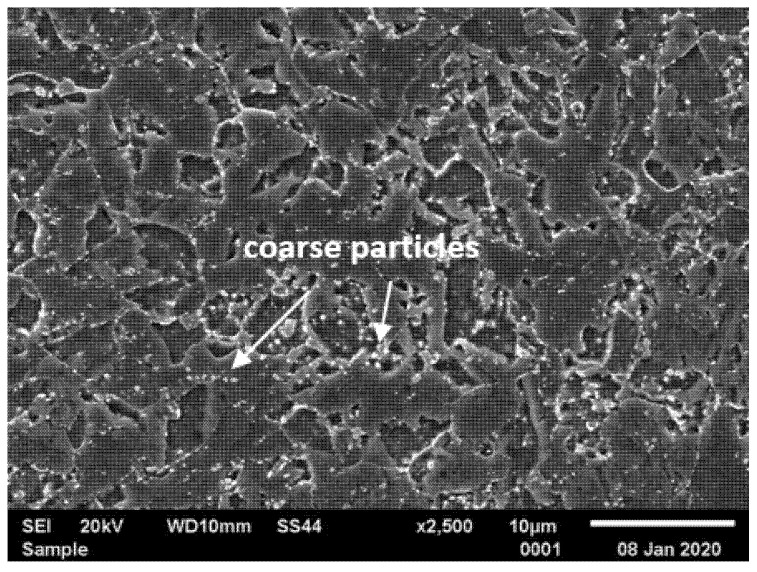
Microstructure of FGHAZ/ICHAZ in T115 steel joint—joint No. 2.

In turn, at the FGHAZ/ICHAZ, the peak temperature which is lower than for CGHAZ—slightly above A_c3_ for FGHAZ and between A_c1_ and A_c3_ for ICHAZ [20,30]. The peak temperature in these areas leads to partial dissolution of precipitates in the matrix [20,32,33]. Undissolved particles inhibit the austenite grain growth by pinning austenite grain boundaries and contributes to the formation of the fine-grained structure (Figure 8 and Figure 9). In both cases, the heat input temperature and the cooling rate are similar to the annealing parameters of the steel. The estimated grain size in FGHAZ was 16 ± 6 µm, which corresponds to grain size of 9 according to the ASTM standard scale. The effect of the thermal cycle temperature in this area may not only contribute to the dissolution of some precipitates in the matrix, but also lead to the coagulation of undissolved M_23_C_6_ carbides [32,33]. Partial dissolution of the particles in this part of the HAZ leads to coarsening of undissolved precipitates. Hence, both large (and small precipitates distributed as in the base material are observed in this area. In this area, a considerable microstructure degradation is observed, and a virtually complete loss of the lath structure of tempered martensite in favour of the ferritic microstructure with numerous particles was visible (Figure 8). 

Similar test results are presented in [31,38]. According to [33], in addition to M_23_C_6_ and MX particles, the Laves phase precipitates are also observed in this area. The formation of Laves phase at grain boundaries reduces the creep properties at the steel as results reduction solid solution strength. The Laves phase also decrease of toughness [39]. In this area, the accelerated recovery of tempered martensite lath structure as compared to the other areas of the joint is observed too [27,34]. A relatively high advancement of the microstructure degradation in these areas was related not only to the effect of the thermal cycle, but also to the treatment of joints following welding. The above changes in the microstructure of this area translate into faster softening compared to the other areas of the joint, resulting in the tendency of this area to the accelerated creep damage, which is called type IV cracking [35]. In joint No. 2, the microstructure in this joint area was similar (Figure 9). 

**Figure 10:**
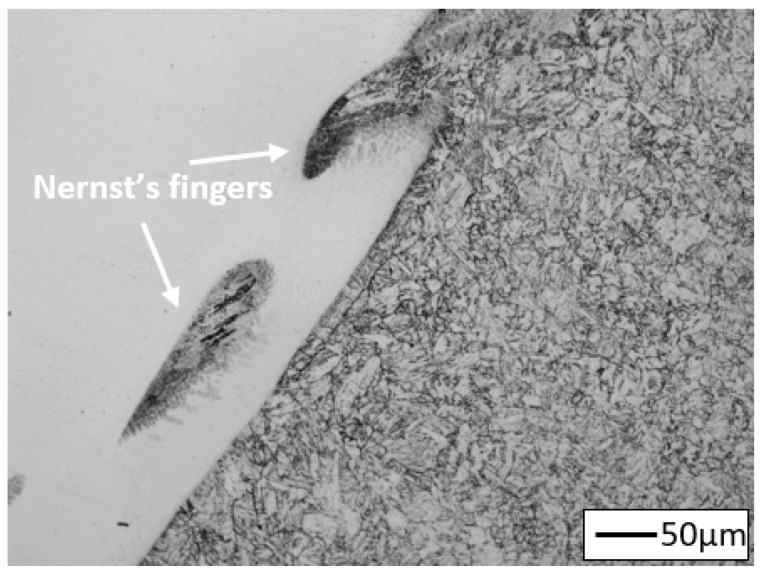
Nernst’s fingers in the weld of joint No. 2.

Moreover, the occurrence of characteristic non-mixed areas between the weld and the base material, i.e., the so-called Nernst’s fingers (NF), was observed at the fusion line in the analysed joint (Figure 10). Their presence in the joint is caused by the existence of a stationary liquid layer in the pool, adjacent to the fusion line, during the welding [9]. Also the formation of the NF occurs mainly due to difference in the melting point of the base material and filler metal [38,40]. Therefore, these areas were not observed in joint No. 1. The detailed mechanism of the formation of NF and its effect on the properties of the joints obtained are presented in [41,42].

### 4.3. Mechanical Properties of Test Joints

**Figure 11:**
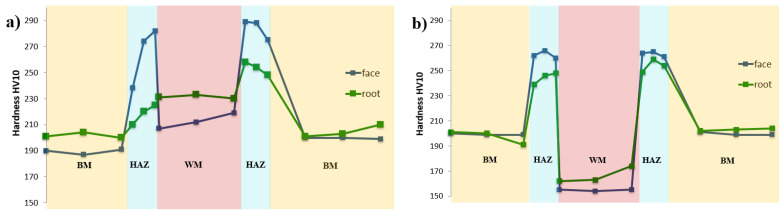
Hardness distribution profile across the cross-section of the joint welded with: (**a**) CrMo91, (**b**) EPRI P87.

**Figure 12:**
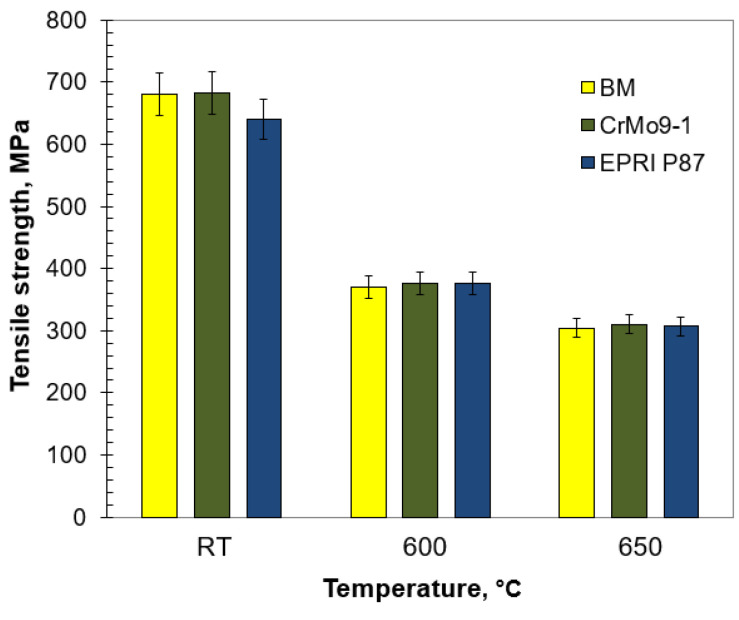
Tensile strength of the test joints welded at a specific temperature.

**Figure 13:**
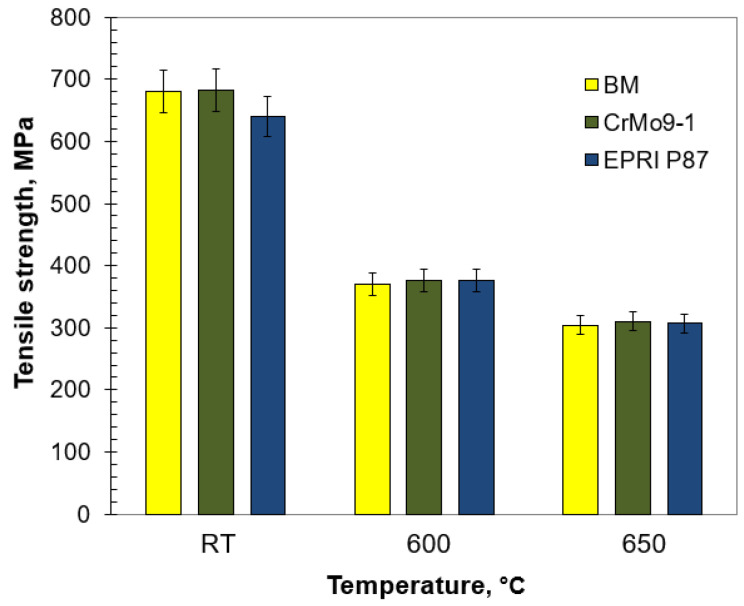
Impact energy of the welded joints for both filler metals.

The results of mechanical investigations of the analysed welded joints are shown in Figure 11, Figure 12 and Figure 13 and Table 5. The mechanical properties of the test joints were referred to the requirements in [43].

The basic investigation carried out on the welded joints No. 1 and No. 2 was hardness measurement taken across the cross-section of the joint. The main factors affecting the hardness of joints in the test steel include: microstructure, fraction area of M_23_C_6_ and MX precipitate, density of dislocations, presence of C and N atoms in solid solution and prior austenite grain size. The measurements showed that the hardness results obtained across the cross-section of the test joints did not exceed the value of 290HV. In the case under consideration, high hardness near the fusion line at the CGHAZ of joint No. 1 is probably due to the precipitation of M_23_C_6_ and MX particles. Heating this area during welding leads to dissolution of M_23_C_6_ carbides and part of vanadium-rich MX precipitates in the matrix [31,32]. The PWHT leads to the precipitation of dispersive particles, resulting in a significant precipitation hardening. In turn, the lower hardness of FGHAZ/ICHAZ compared to CGHAZ results from the coagulation of M_23_C_6_ carbides and more advanced matrix softening during the PWHT [20]. The differences in microhardness in the vicinity of the fusion line between joint No. 1 and joint No. 2 are probably caused by the presence of a soft δ-ferrite patch in the microstructure of joint No. 2 (Figure 11, Table 5). The impact of the δ-ferrite on the reduction in hardness of weld fusion zone is also presented by Pandey [31,34]. Poor mechanical properties of δ-ferrite confirm the poor resistance to deformation and affects the tendency to failure during creep service [33]. Hence formation of δ-ferrite in the HAZ of the steel should be avoided. The observed differences in weld material hardness in the test joints are the result of using filler materials with different chemical compositions. This results in different microstructures in the weld area—martensitic (joint No. 1) or austenitic (joint No. 2) microstructure.

Moreover, the measurements (Table 5) showed that the lowest value of microhardness across the cross-section of the joint was observed in the FGHAZ/ICHAZ. Low microhardness of this soft zone resulted from the advanced degradation of microstructure of this zone (Figure 8 and Figure 9) and was related to the softening of martensite (recovery process) and growth and the coarsening of the precipitation [34,35]. Low hardness/microhardness in this area shows the high degree of degradation of the microstructure. The high degree of structure degradation in this area and its lower properties translate into the increase in its susceptibility to Type IV cracking [32,35].

The static tensile test of welded joints was carried out at both room and elevated temperatures. The tensile strength values of the analysed joints were higher than the required minimum for the base material (Figure 12). Regardless of the temperature, the TS values for the test joints were similar to each other and comparable to those obtained for the base material. Nevertheless, TS at 600 and 650 °C was slightly less than half of the TS at room temperature. Due to the possibility of faster diffusion, the elevated temperature makes the movement of dislocation easier and allows precipitations to be avoided by climbing [44]. This translates into decreasing interaction between precipitates and matrix, therefore the precipitate strengthening mechanism gradually weakened and the tensile strength subsequently decreased. According to Xiao et al. [45], this phenomenon might be related to subgrain coarsening and decreasing dislocation density. The comparable TS values of the analysed joints regardless of the temperature indicate a slight impact of the δ-ferrite on this strength parameter. The rupture of the analysed samples took place in the base material. 

In creep-resistant ferritic/martensitic steels, high impact energy is used as a major criterion to ensure the material resistance towards the embrittlement and increase of ductile–brittle transaction temperature. Due to the presence of various areas in the HAZ, the impact energy of the welded joint will vary. The minimum impact energy of the steel weldments was recommended to be about 52 J/cm^2^ as per [43]. The results of the impact test performed on the test joints, which are the measure of the material’s capability of transferring dynamic loads, are summarised in Figure 13. Generally speaking, the impact energy of metallic materials depends mainly on the grain size (structure refinement), but also on the precipitate morphology and presence of brittle phases at the grain boundaries. Regardless of the filler metal used, the impact strength of the welded joints was higher than that required for the base material. The lower impact energy of HAZ in joint No. 2 is probably due to the presence of the δ-ferrite patch with carbides at the grain boundaries in the vicinity of the fusion line (Figure 7). Particles precipitated at the δ-ferrite/martensite interphase boundary lead to poor cohesions between the PAGs and the δ-ferrite [33,35]. However, the effect of the δ-ferrite patch on toughness and ductility is ambiguous. Some researchers concluded its negative impact [33,46], while others said it was positive [10]. According to Moon [46] impact energy of tempered martensite is superior to those of martensite and δ-ferrite. The higher impact energy of HAZ in joint No. 1 resulted from the homogeneous microstructure deprived of the δ-ferrite (Figure 6). Similarly to the tensile strength, the differences in impact energy of the weld were caused by the filler metal used for welding (Table 3 and Table 4). In case of joint No. 2, it was the austenitic filler characterised by high toughness and ductility at relatively low strength properties (Table 3 and Table 4).

## 5. Conclusions

The investigations of microstructure and mechanical properties were carried out on T115 steel joints welded with two filler metals: CrMo91 (No. 1) and EPRI P87 (No. 2). They allowed the following statements and conclusions to be put forward:

1. Microstructure of HAZs in the analysed joints is typical of this group of steels. It was shown that the HAZ of joint No. 1 had a homogeneous tempered martensite microstructure without presence of the δ-ferrite, whereas the δ-ferrite patch was observed in the vicinity of the fusion line of CGHAZ in joint No. 2.
2. The presence of the δ-ferrite in CGHAZ of joint No. 2 had a slight influence on the tensile strength and hardness of the analysed joints and a significant effect on the impact energy of the HAZ.
3. In both of the analysed joints, the presence of the FGHAZ/ICHAZ with significant microstructure degradation was revealed, which was reflected in the minimum microhardness compared to the other areas of the joint.
4. Mechanical testing of the sample Thor 115 steel joints welded with both filler materials (CrMo91 and EPRI P87) confirmed good strength properties and good resistance to dynamic loads.
5. The results obtained from investigations confirmed a high quality of the joints, good weldability of T115 steel and demonstrated that the filler metals used met the strength and quality requirements.

## Figures and Tables

**Table 1:** Chemical composition of the Thor 115 steel, wt.%.

C	Mn	Si	Cr	Mo	Ni	Cu	V	Nb	N
0.09	0.47	0.15	11.30	0.52	0.16	0.08	0.24	0.04	0.002

**Table 2:** Mechanical properties of T115 steel in as-received condition.

	Mechanical Properties of T115 Steel			
YS (MPa)	TS (MPa)	El. (%)	KV (J)	HV30
610	687	27	158	220

**Table 3:** Chemical composition of filler materials, % wt.

Filler Material	C	Si	Mn	Cr	Mo	Nb	V	Ni	Fe
CrMo91	0.09	0.26	0.45	9.20	0.91	0.052	0.21	0.41	bal.
EPRI P87	0.11	0.16	1.55	8.52	2.02	1.09	-	bal.	38.8

**Table 4:** Mechanical properties of CrMo91 and EPRI P87 filler materials.

	Mechanical Properties of T115 Steel			
Filler material	YS MPa	TS MPa	KV J	HV30
CrMo91	690	780	150	-
EPRI P87	360	560	32	150

**Table 5:** Results of microhardness measurement of the test welded joints and δ-ferrite.

Location of Measurement		Joint No.	
		1 (WCrMo91)	2 (EPRI P87)
		HV0.1	
WM	face	234–244	124–143
	root	237–247	173–180
CGHAZ		273–285	232–245
FGHAZ/ICHAZ		191–207	185–206
BM		212–216	
δ-ferrite		145–168 *	

*—HV0.001.

## Data Availability

The data presented in this study are available on request from the corresponding author.
